# Atypical exuberant presentation of sycosiform tinea barbae

**DOI:** 10.1590/0037-8682-0444-2021

**Published:** 2021-12-17

**Authors:** Valentina Lourenço Lacerda de Oliveira, Emilly Neves Souza, Lucia Martins Diniz, Luana Amaral de Moura

**Affiliations:** 1 Universidade Federal do Espírito Santo, Programa de Residência Médica em Dermatologia, Vitória, ES, Brasil.; 2 Universidade Federal do Espírito Santo, Departamento de Clínica Médica (Dermatologia), Vitória, ES, Brasil.

**FIGURE 1:**
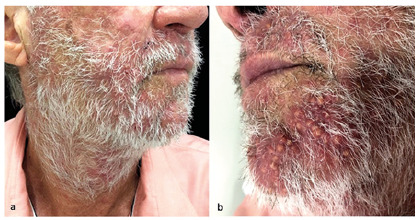
**(a)** Erythematous papules on the right malar and erythematous nodular-cystic lesions in the beard area. **(b)** Erythematous papules and erythematous nodular-cystic lesions in the beard area.

**FIGURE 2:**
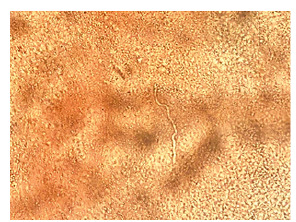
Direct mycological examination with KOH 20% revealed the presence of hyaline and septate dermatophyte hyphae.

**FIGURE 3:**
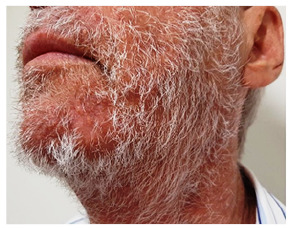
Regression of skin lesions 30 days after treatment.

A 71-year-old man with cirrhosis was hospitalized for hepatic encephalopathy and developed pruritic facial papules. The lesions were considered bacterial folliculitis, which initially emerged on the upper lip and spread to the beard area. The patient was prescribed systemic antibiotics to treat the lesions. The lesions were unresponsive to the treatment. The dermatology team was requested to observe yellowish erythematous papules and nodular-cystic lesions on the face, especially the beard area (Figure 1). The patient also exhibited erythematous violaceous plaques covered by pustules and superficial desquamation on the left forearm, as well as brownish circinate inguinal and gluteal macules. Incisional biopsies were performed on the patient’s face and forearm. The anatomopathological findings indicated acute suppurative inflammatory folliculitis with intense macrophage reaction. Direct mycological examination revealed septate and branched hyaline hyphae (Figure 2). Anatomopathological and mycological studies diagnosed sycosiform tinea barbae. After treatment with terbinafine for thirty days, clinical improvement was seen in the patient (Figure 3).

Tinea barbae is an exclusive male dermatophytosis that is seen commonly in young adults^1^. Bacterial or viral folliculitis is usually the first diagnostic hypothesis^1^ ^,^ ^2^ of this condition which leads to misdiagnosis. This can lead to inappropriate antibiotic use, as seen here, exposing patients to potential side-effects and delaying the correct treatment.

The diagnosis is confirmed by direct examination, cultures, and microcultures of the lesion samples^1^ ^),(^ ^3^. Treatment options include griseofulvin and terbinafine^3^. Although occurrences of sycosiform tinea barbae is uncommon in older adults, healthcare professionals should be able to detect this dermatophytosis to avoid misdiagnosis.
